# Radiotherapy in oligometastatic prostate cancer—a pattern of care survey among members of the German Society for Radiation Oncology (DEGRO)

**DOI:** 10.1007/s00066-022-01925-2

**Published:** 2022-04-01

**Authors:** Paul Rogowski, Christian Trapp, Rieke von Bestenbostel, Dinah Konnerth, Sebastian Marschner, Nina-Sophie Schmidt Hegemann, Claus Belka, Minglun Li

**Affiliations:** 1 Department of Radiation Oncology, University Hospital, LMU Munich, Marchioninistr. 15, 81377 Munich, Germany; 2 German Cancer Consortium (DKTK), Munich, Germany

**Keywords:** OMPC, Primary-directed therapy, Metastasis-directed therapy, ENRT, PSMA-PET/CT

## Abstract

**Purpose:**

Due to improved imaging, oligometastatic prostate cancer (OMPC) is diagnosed more frequently. Growing evidence shows that patients with a limited number of metastases benefit from primary-directed radiotherapy (PDT) as well as from metastasis-directed radiotherapy (MDT). This survey investigates the current treatment practice for OMPC among German-speaking radiation oncologists.

**Methods:**

Members of the German Society for Radiation Oncology (*Deutsche Gesellschaft für Radioonkologie* [DEGRO]) were surveyed regarding their current treatment practice via an anonymous online questionnaire sent by email. The survey included six general items and 14 specific items regarding treatment characteristics. Questionnaires with at least 50% of questions completed were considered for further analysis.

**Results:**

A total of 204 responses were received (15% response rate), 167 were considered for further analysis. Most respondents stated to be specialized in treating prostate cancer patients and to treat 10–30 patients with OMPC per annum; 97% considered PSMA-PET/CT necessary to define oligometastatic disease. Opinions differed regarding the use of systemic therapies: 63% of the respondents aimed to defer systemic therapy using radiotherapy in OMPC, whereas 37% considered systemic therapy necessary. In the setting of synchronous OMPC, 97% recommended PDT with or without a combination of MDT and/or systemic therapy. For metachronous nodal or bone oligometastatic recurrence, 98 and 99%, respectively, would opt for MDT. The majority would combine MDT with systemic therapy in patients with metachronous oligorecurrence. Respondents recommended normofractionation, hypofractionation, and SBRT for lymph node metastases in 49, 27, and 24%, respectively. No consensus existed regarding the field size for MDT of lymph node metastases. Most respondents preferred > 5 fractions for treatment of bone metastases.

**Conclusion:**

Local radiotherapy for PDT and MDT is routinely used among respondents of this survey, representing 12% of all German-speaking radiation oncologists. The timing of systemic therapy, fractionation schedules, and field sizes are handled differently and remain an area of active investigation.

## Background

In 1995, Hellman and Weichselbaum introduced the term oligometastatic cancer for a state between localized and widespread disease, which is amenable to local treatment [1]. Sensitive PSA detection and improved imaging are increasingly leading to diagnosis of oligometastatic prostate cancer (OMPC) [2], and growing evidence shows that prostate cancer (PCa) patients with a limited number of metastases benefit from primary-directed therapy (PDT). In the randomized phase III Systemic Therapy in Advancing or Metastatic Prostate Cancer: Evaluation of Drug Efficacy (STAMPEDE) trial, local treatment of the primary improved overall survival in patients with a low metastatic burden [3]. Metastasis-directed therapy (MDT) is still a matter of debate. However, several phase II trials have reported favorable outcomes with MDT in OMPC. Palma and colleagues showed a survival benefit for stereotactic body radiotherapy (SBRT) to all metastatic lesions [4]. However, this study included patients across various histologies of which only 27% were PCa. The Observation vs Stereotactic Ablative Radiation for Oligometastatic Prostate Cancer (ORIOLE) trial reported improved biochemical progression-free survival in the entire study population and prolonged distant metastasis-free survival in a subgroup in which all prostate-specific membrane antigen positron-emission tomography/computed tomography (PSMA-PET/CT)-positive lesions were treated [5]. Moreover, Ost et al. investigated the deferral of androgen deprivation therapy (ADT) by MDT and established androgen deprivation therapy-free survival (ADT-FS) as a new endpoint [6]. MDT prolonged ADT-FS from 13 to 21 months compared to surveillance.

On the basis of data from the STAMPEDE trial, current national and international guidelines recommend PDT for patients with newly diagnosed OMPC additional to systemic treatment [7, 8]. The current German guideline states that MDT is not supported by sufficient evidence with regard to oncological outcomes but can be used to defer ADT and tumor progression. The European guideline recommends metastasis-directed therapy to metastatic PCa patients only within clinical trials or well-designed prospective cohort studies. Nevertheless, many vital questions in the treatment of OMPC are pending. In particular, the role of imaging methods, the relevance of the site of metastasis, combinations with systemic therapies, radiation fractionation schedules, and the optimal field size. There is also growing evidence that different subcategories of oligometastasis should be distinguished [9, 10].

The purpose of this anonymous online survey was to investigate the current treatment practice for OMPC among German radiation oncologists.

## Methods

An anonymous, web-based survey was developed with the online survey tool LimeSurvey (LimeSurvey GmbH, Hamburg, Germany) licensed for use by the Ludwig Maximilian University of Munich. The survey contained 6 questions regarding respondent and institution characteristics and 14 specific questions regarding radiotherapy practice for OMPC (Tables 1 and 2). Participants were instructed to choose answers from a multiple-choice questionnaire allowing for one or, in some cases, multiple answers. A link to the questionnaire was sent via email to approximately 1361 radiation oncologists compiled through the *Deutsche Gesellschaft für Radioonkologie e.* *V.* (DEGRO) directory, the official German Society for Radiation Oncologists.

**Table 1:** Respondents and institutions characteristics

*Years of experience in radiooncology*	
< 5 years	7.2%
5–10 years	12.0%
> 10 years	80.8%
*Type of facility*	
Academic center (university hospital)	22.2%
Nonacademic hospital	21.6%
Private practice/ambulatory healthcare center	56.3%
*Specific expertise in treating PCa*	
Yes	77.8%
No	12.6%
No answer	9.6%
*Are urooncological patients in your facility usually discussed in a multidisciplinary tumor board?*	
Yes	79.6%
No	20.4%
*Number of OMPC patients treated per annum*	
< 10	28.1%
10–30	57.5%
> 30	13.8%
No answer	0.6%
*Number of OMPC patients treated with PDT*	
< 10	43.7%
10–30	42.5%
> 30	12.6%
No answer	1.2%
*Number of OMPC patients treated with MDT*	
< 10	29.9%
10–30	52.1%
> 30	16.8%
No answer	1.2%

*MDT* metastasis-directed therapy, *OMPC* oligometastatic prostate cancer, *PCa* prostate cancer, *PDT *primary-directed therapy

**Table 2:** Radiotherapy characteristics

*Which staging method do you consider necessary to define oligometastasis in PCa (multiple selection possible)?*	
Choline- or PSMA-PET/CT	97.0%
CT of the thorax and abdomen	29.9%
Bone scan	29.3%
MRI of the abdomen and pelvis	28.1%
Whole-body MRI	4.8%
*How is oligometastasis defined in your facility?*	
1 metastatic lesion	1.8%
≤ 2 metastatic lesions	3.6%
≤ 3 metastatic lesions	34.1%
≤ 4 metastatic lesions	4.8%
≤ 5 metastatic lesions	28.1%
If a curative-intent therapy to all lesions is safely possible	25.7%
I do not believe that oligometastasis exists as a separate stage between localized and metastatic disease	1.2%
No answer	0.6%
*Which of the following statements best corresponds to your opinion on systemic therapy for OMPC?*	
Systemic therapy is always recommended for metastatic prostate cancer	37.1%
Radiotherapy should aim to defer systemic therapy	62.9%
*Which treatment would you recommend in a fit patient with synchronous oligometastasis and an untreated primary?*	
Systemic treatment only	0%
PDT only	1.2%
PDT and systemic treatment	3.0%
MDT only	0.6%
MDT and systemic treatment	2.4%
PDT and MDT	29.3%
PDT and MDT and systemic treatment	63.5%
*Which fractionation would you choose for PDT?*	
Normofractionation	57.5%
Hypofractionation	37.7%
SBRT	4.8%
*Does the indication for MDT differ in OMPC depending on whether it is distant lymph node metastases or bone metastases?*	
Yes	58.7%
No	41.3%
*Which treatment would you recommend in a fit patient with oligometastatic recurrence with bone metastases?*	
Systemic treatment only	1.2%
MDT only	44.9%
MDT and systemic treatment	53.9%
*Which fractionation do you prefer for MDT for bone metastases?*	
< 3 fractions	4.2%
3–5 fractions	29.9%
> 5 fractions	65.9%
*Which treatment would you recommend in a fit patient with oligometastatic recurrence with distant lymph node metastases (M1a)?*	
Systemic therapy only	1.2%
Metastasis-directed radiotherapy (MDT) only	37.7%
MDT + systemic therapy	60.5%
No answer	0.6%
*Which fractionation would you prefer for MDT for distant lymph node metastases (M1a)?*	
Normofractionation	49.1%
Hypofractionation	26.9%
SBRT	24.0%
*Which field size would you prefer for MDT for distant lymph node metastases (M1a)?*	
Focal irradiation of the affected lymph node (involved-node radiotherapy)	33.5%
Irradiation of the affected lymph node region (involved-field radiotherapy)	22.8%
Irradiation of the affected and adjacent lymph node regions (elective nodal radiotherapy)	29.9%
Additional inclusion of the entire pelvic lymphatic drainage area (whole-pelvic radiotherapy)	13.2%
No answer	0.6%
*Do you differentiate in the treatment with MDT between synchronous and metachronous oligometastatic disease?*	
Yes	43.1%
No	56.3%
No answer	0.6%
*Which of these factors do you consider relevant for the decision for or against radiotherapy for oligometastatic prostate cancer (multiple selection possible)?*	
Age	61.7%
Number of metastases	98.8%
Gleason Score	45.5%
Initial PSA	25.1%
PSA before radiotherapy	38.3%
PSA doubling time	61.1%
Hormone sensitivity	57.5%

*CT* computed tomography, *MDT* metastasis-directed therapy, *MRI* magnetic resonance imaging, *PCa* prostate cancer, *PDT* primary-directed therapy, *PSA* prostate specific antigen, *PSMA-PET/CT* prostate specific membrane antigen positron-emission tomography/computed tomography, *SBRT* stereotactic body radiotherapy

The email invitation containing rationale, instructions on participation and contact information was sent out on May 19, 2021, with a reminder ensuing on June 9, 2021, to maximize response rate. Responses were collected from May to July 2021. Because the questionnaire allowed to skip single or multiple questions without answering, only those questionnaires with at least 50% of the questions answered were eligible for analysis using descriptive statistics. Ethical approval for a pattern of care study comprising an anonymous online questionnaire was not applicable.

## Results

A total of 204 responses were received (15% response rate). Of these, 167 respondents (81.4%) had answered at least 50% of the questions, and these questionnaires were hence considered for further analysis. Characteristics of the respondents and institutions are summarized in Table 1. Most respondents were experienced radiation oncologists with more than 10 years of experience (80.8%) and had specific expertise in treating PCa (77.8%). Respondents worked in public academic facilities, nonacademic facilities, and private practices in 22.2, 21.6, and 56.3%, respectively. The majority of respondents reported to treat < 10 patients annually with PDT (43.7%) and 10–30 patients with MDT (52.1%).

Table 2 shows the 14 items addressing treatment characteristics and associated responses. The large majority of respondents (97.0%) deemed staging with PSMA-PET/CT necessary for definition of oligometastatic disease. Oligometastatic disease was most often defined by the respondents as ≤ 3 (34.1%) and ≤ 5 metastatic lesions (28.1%), and as a disease state with the possibility to safely treat all lesions with curative-intent therapy (25.7%). Opinions differed with regard to systemic therapy: 62.9% of respondents answered that radiotherapy should aim to defer systemic therapy in OMPC, whereas 37.1% considered systemic therapy to always be necessary in metastatic disease. Nevertheless, most respondents recommended PDT plus MDT and systemic therapy for a fit patient with synchronous oligometastasis and an untreated primary (63.5%). PDT with or without a combination of MDT and/or systemic therapy was recommended in this case by 97.0% of respondents. None of the respondents opted for systemic therapy only in this setting. Normofractionation, hypofractionation, and SBRT for PDT would have been chosen by 57.5, 37.7, and 4.8%, respectively.

With regard to the indication for MDT, most of the respondents did not distinguish between synchronous and metachronous oligometastatic disease (56.3%) but made a distinction between distant lymph node metastases and bone metastases (58.7%). In case of a fit patient with bone oligorecurrence, 53.9% recommended MDT with systemic therapy and 44.9% recommended MDT only. In case of distant lymph node oligorecurrence, MDT with and without systemic therapy was recommended by 60.5 and 37.7%, respectively. Most respondents would choose a fractionation scheme with > 5 fractions for MDT for bone metastases (65.9%). For MDT of distant lymph node oligorecurrence, 49.1% of respondents would offer normofractionated radiotherapy, 26.9% hypofractionated radiotherapy, and 24.0% stereotactic body radiotherapy (SBRT). Regarding the field size for MDT in the case of distant lymph node oligorecurrence, most respondents recommended involved-node radiotherapy (INRT; 33.5%), followed by elective nodal radiotherapy (ENRT; 29.9%), involved-field radiotherapy (IFRT; 22.8%), and additional inclusion of the entire pelvic lymphatic drainage area (whole-pelvic radiotherapy; 13.2%).

The parameters considered by respondents as most relevant for the decision for radiotherapy in OMPC were number of metastases (98.8%), age (61.7%), prostate-specific antigen (PSA) doubling time (61.1%), and hormone sensitivity (57.5%). Gleason score (45.5%), PSA value before radiotherapy (38.3%), and initial PSA value (25.1%) were considered less important.

## Discussion

While OMPC is recognized as a special oncologic setting with a growing body of encouraging evidence for effective local treatment of primary and metastases, the answers to many vital questions are still pending [11]. Nevertheless, local therapies have found their way into the clinical practice of radiation oncologists. Therefore, this survey aimed to highlight the treating practice for OMPC among DEGRO members. The majority of respondents declared in the questionnaire to have more than 10 years of experience and to be specialized in the treatment of PCa. As expected, the respondents came from academic and nonacademic hospitals and approximately half of them claimed to work in private practices.

Staging with PSMA-PET/CT is increasingly applied in primary and recurrent PCa and has been shown to outperform every other imaging technique [12, 13]. Accordingly, the vast majority of respondents of this survey considered choline- or PSMA-PET/CT as necessary to define oligometastatic disease over any other imaging modality. However, the definition of OMPC in the literature is inconsistent, and it is thus not surprising that respondents defined OMPC with different numerical cutoffs ranging from one to five metastases. The STAMPEDE trial showed that prostate radiotherapy improved survival in patients with low but not with high metastatic burden (defined according to ChemoHormonal Therapy Versus Androgen Ablation Randomized Trial for Extensive Disease in Prostate Cancer (CHAARTED) criteria) [3, 14]. Furthermore, in a secondary analysis by Ali and colleagues, the benefit of PDT was greater in patients with nonregional lymph nodes (M1a) or up to three bone metastases than among patients with four or more bone metastases or visceral metastases [15]. Of note, in the STAMPEDE trial, metastatic burden was assessed with bone scans, MRI, and CT, which have been shown to have lower detection rates than PSMA-PET/CT. Therefore, patients with an even higher metastatic burden in PSMA-PET/CT might also benefit from local treatment, making a strict numerical cutoff difficult. Noteworthily, a relevant proportion of respondents of this survey defined oligometastatic disease as a disease state in which a curative-intent treatment to all metastases is possible. This might be a reasonable approach as long as there is neither a precise numerical definition nor molecular markers for the oligometastatic state in PCa.

In metastatic hormone-sensitive PCa, androgen deprivation therapy in combination with novel hormonal agents like abiraterone, enzalutamide, and apalutamide or chemotherapy with docetaxel is the standard of care. However, these therapies can be associated with adverse effects and a deterioration in quality of life. In this survey, two thirds of respondents agreed that the aim of radiotherapy in oligometastatic disease should be to delay systemic therapy, probably owing to evidence from trials investigating a controversial approach of using local therapies in OMPC to defer systemic therapy [5, 6]. Remarkably and partially in contrast to the previous statement, in the given setting with synchronous OMPC, 69% of the respondents in this survey favored a combination of systemic therapy and local radiotherapy. In the setting of metachronous nodal or bone oligometastatic recurrence, still 61 and 54%, respectively, would opt for a combination with systemic therapy. Nevertheless, the approach using local radiotherapies in OMPC to defer systemic therapy should be used with caution since subclinical disease is missed by imaging in a significant proportion of oligometastatic patients and delaying systemic therapy may therefore reduce long-term tumor control. Indeed, also the majority of panelists of the Advanced Prostate Cancer Consensus Conference (APCCC) 2019 voted for adding MDT to systemic therapies, instead of it replacing them [16].

Our findings show that the optimal dose schedule and the optimal field size for radiotherapy in OMPC remain controversial. For PDT, the majority of respondents in the survey would recommend a normofractionated (58%) or hypofractionated treatment (38%). No precise cutoff values for hypofractionation and SBRT (which is commonly used synonymously with ultrahypofractionation) were provided in the questionnaire. Typically, doses between 2.2 Gy and 4 Gy are regarded as hypofractionated and doses of 4 Gy and beyond as ultrahypofractionated [17]. The best evidence exists for the two schedules used in the STAMPEDE trial (55 Gy in 20 fractions over 4 weeks or 36 Gy in 6 fractions over 6 weeks). Of note, a prespecified analysis found heterogeneity in the effect on failure-free survival by the two fractionation schemes. While the daily schedule led to a clear advantage in failure-free survival, the effect in patients with the weekly schedule was less pronounced. However, in designing the STAMPEDE trial, less burdensome concepts compared to conventional fractionation schedules for definitive prostate radiotherapy were intentionally chosen for metastatic disease. Thus, radiotherapy with sufficient dose, normofractionated or hypofractionated, may further improve oncological outcomes.

With regard to MDT, the heterogeneity of radiotherapeutic approaches is even greater. First, a distinction can be made between distant lymph node and bone metastases, as the prognosis in lymph node-positive PCa has proved to be superior [18, 19]. In this survey, 59% of respondents stated that their indication for radiotherapy depended on whether it was distant lymph node or bone oligometastatic disease. Due to the rarity of occurrence, other sites of distant metastasis were not included in the questionnaire. Although so far there is no grade 3 evidence for MDT, almost 99% of respondents in this survey recommended MDT in both settings. Furthermore, more respondents opted for a combination with systemic therapy in the case of lymph node metastases compared to bone metastases (61 vs. 54%). Systemic therapy alone was recommended only by 1.2%.

For MDT of lymph node metastases, several approaches have been suggested, ranging from focal INRT with SBRT to IFRT, ENRT, and whole-pelvis radiotherapy [20–22]. Our results show that the optimal field size is still very controversial and that each of these approaches are favored by a relevant number of respondents. In the literature, SBRT and ENRT were directly compared in a large retrospective analysis by De Bleser and colleagues. They analyzed 506 patients with nodal oligorecurrence staged mostly by choline PET/CT and treated by either ENRT (with or without SIB) or SBRT. Patients had a significantly improved 3‑year metastasis-free survival of 77% when treated with ENRT compared to 68% after SBRT. However, early and late toxicity was higher in the ENRT group [23]. Lepinoy et al. retrospectively compared IFRT, mostly using 36 Gy in 5 fractions, to conventionally fractionated whole-pelvis radiotherapy. Again, the use of extended fields was associated with a significantly longer failure-free time, albeit at the cost of more acute gastrointestinal toxicity [24]. Of note, survival data are still lacking.

Regarding the treatment of bone oligometastases, respondents favored various fractionation schedules ranging from SBRT in one or two fractions to regimens with more than five fractions. This reflects the ongoing debate in the literature regarding the best dose concepts in bone OMPC [25]. Retrospective data have suggested better oncological outcomes with BED_3_ > 100 Gy [26, 27]. However, series with lower BED_3_ reported very good local control rates as well [10].

Recently, a European Society for Radiotherapy and Oncology (ESTRO) and European Organisation for Research and Treatment of Cancer (EORTC) consensus recommendation suggested a classification of oligometastatic disease with the goal to better differentiate between various subcategories, i.e., synchronous and metachronous disease [28]. Also, the majority of panelists of APCCC considered it important to distinguish between these two categories [16]. In our survey, 56% of the respondents stated to make no distinction between synchronous and metachronous OMPC. This may be due to the inconsistent data in the literature. While some retrospective data show better oncological outcomes in synchronous OMPC compared to metachronous disease, other studies report contradicting results [10, 24, 29, 30].

The major limitation of our study is the low response rate of 15%, with a sample size of 167 responses considered for analysis. Moreover, the nature of surveys itself can lead to biases because respondents might describe what they would like to see in daily professional work in their answers, rather than the answers mirroring current practice in the respective institution. Selection bias could not be avoided, as more interested radiation oncologists were more likely to participate in this survey. Furthermore, respondents might tend to use radiotherapy in OMPC more often than the majority of the nonrespondents. Thus, our findings have to be interpreted with caution, as they may not be representative of other colleagues who chose not to participate. Nevertheless, to the best of our knowledge, the study represents the first survey among German radiation oncologists on this important issue. We believe our survey has added important insight into the treatment practice of OMPC and may serve as a basis for future prospective studies. An overview of open questions to be addressed in future clinical trials is presented in Table 3.

**Table 3:** Overview of open questions to be addressed in future clinical trials

What is the optimal systemic therapy in combination with PDT for synchronous OMPC?
What is the recommended field size for PDT (inclusion of pelvic RT for N1 OMPC)?
What is the optimal fractionation schedule for PDT?
What is the effect of MDT to all lesions in synchronous OMPC (in addition to PDT and systemic therapy)?
Does MDT provide an OS benefit in OMPC?
Can MDT defer systemic therapy without compromising OS?
What is the optimal fractionation schedule for MDT?
What is the optimal field size for MDT of lymph node metastases?

*MDT* metastasis-directed therapy, *OMPC* oligometastatic prostate cancer, *OS* overall survival, *PDT* primary-directed therapy, *RT* radiotherapy

## Conclusion

This survey provides real-life data on the pattern of care for OMPC among German radiation oncologists. Radiotherapy for PDT and MDT is routinely used among respondents, representing 12% of all German-speaking radiation oncologists. The timing of systemic therapy, fractionation schedules, and field sizes are handled differently and remain an area of active investigation. Prospective trials are warranted to investigate the optimal treatment strategy for OMPC.

## Abbreviations

ADT: Androgen deprivation therapy
ADT-FS: Androgen deprivation therapy-free survival
BED3: Biological effective dose with an alpha/beta value of 3 Gy
CT: Computed tomography
DEGRO: *Deutsche Gesellschaft für Radioonkologie e.* *V.* (German Society for Radiation Oncology)
ENRT: Elective nodal radiotherapy
EORTC: European Organisation for Research and Treatment of Cancer
ESTRO: European Society for Radiotherapy and Oncology
IFRT: Involved-field radiotherapy
INRT: Involved-node radiotherapy
MDT: Metastasis-directed therapy
MRI: Magnetic resonance imaging
OMPC: Oligometastatic prostate cancer
PCa: Prostate cancer
PDT: Primary-directed therapy
PSA: Prostate-specific antigen
PSMA-PET/CT: prostate-specific membrane antigen positron-emission tomography/computed tomography
SBRT: Stereotactic body radiotherapy
